# Robot Trajectories Comparison: A Statistical Approach

**DOI:** 10.1155/2014/298462

**Published:** 2014-11-25

**Authors:** A. Ansuategui, A. Arruti, L. Susperregi, Y. Yurramendi, E. Jauregi, E. Lazkano, B. Sierra

**Affiliations:** ^1^Autonomous and Smart Systems Unit, IK4 Tekniker, Eibar, Spain; ^2^Department of Computation and Artificial Intelligence, Euskal Herriko Unibertsitatea UPV-EHU, Donostia, Spain

## Abstract

The task of planning a collision-free trajectory from a start to a goal position is fundamental for an autonomous mobile robot. Although path planning has been extensively investigated since the beginning of robotics, there is no agreement on how to measure the performance of a motion algorithm. This paper presents a new approach to perform robot trajectories comparison that could be applied to any kind of trajectories and in both simulated and real environments. Given an initial set of features, it automatically selects the most significant ones and performs a statistical comparison using them. Additionally, a graphical data visualization named polygraph which helps to better understand the obtained results is provided. The proposed method has been applied, as an example, to compare two different motion planners, FM^2^ and WaveFront, using different environments, robots, and local planners.

## 1. Introduction

The task of planning a collision-free trajectory from a start to a goal position is fundamental for an autonomous mobile robot. Although path planning has been extensively investigated since the beginning of robotics, there is no agreement on how to measure the performance of a motion algorithm. Measuring the performance of a motion planner is not an easy task. On the one hand, the existing motion planning approaches are different in nature and their implementations heavily depend on the underlying environment representation. Whether a motion planner performs well or not depends on a large number of factors, often related with the task for which the method is being used. On the other hand, another issue when evaluating a motion planner is that robot motion task is an active task: it is not possible to collect logs and then run algorithms off-line, since the decisions taken by the algorithm in each step determine a new situation and thus new sensor readings and possibilities.

Many scientific disciplines take advantage of widely accepted comparison methodologies, performance metrics, and benchmark databases. Some examples are the PASCAL Object Recognition Database Collection for vision (http://pascallin.ecs.soton.ac.uk/challenges/VOC/databases.html), UC Irvine Machine Learning repository (http://archive.ics.uci.edu/ml/) for the empirical analysis of machine learning algorithms, and Radish (http://radish.sourceforge.net/) (Robotic Data Set Repository), focused on localization and mapping. It is of vital importance for any evaluation framework to identify some performance metrics, define a comparison methodology, and offer a set of benchmark problems.

In this paper, we present a method to compare different navigation approaches, with the aim of helping decide which of them would be better for the purposes in which the robot is to be used. The approach we present is mainly based on statistical measures for the trajectories performed and automatically selects the most significant features among a set of initial features to measure the goodness of a trajectory. The proposed method is used to compare two motion planners, the Fast Marching Square (FM^2^) planner [1] and the WaveFront planner [2]. The FM^2^ planner has been integrated as a plug-in driver in the Player/Stage multirobot simulation tool [3] and compared with the already existing WaveFront planner. The algorithms have been applied under the same conditions and tested in different environments and for two different robots combined with two local planners.

The rest of the paper is organized as follows: after reviewing the related works (Section 2), Section 3 focuses on the proposed approach; Section 4 is devoted to the experimental setup; obtained experimental results are shown in Section 5, and finally in Section 6 the conclusions are presented and the future work lines are pointed out.

## 2. Related Works

The field of robot motion planning has been an active area of research over the last forty years, yielding many motion planning approaches. For example, Berg et al. present LQG-MP (linear-quadratic Gaussian motion planning) [4], a new approach to robot motion planning that takes into account the sensors and the controller that will be used during the execution of the robots path. LQG-MP is based on the linear-quadratic controller with Gaussian models of uncertainty and explicitly characterizes in advance (i.e., before execution) the a priori probability distributions of the state of the robot along its path. Wei and Liu [5] present a novel evolutionary G3continuous (continuous-differentiable curvature) path planner for nonholonomic wheeled mobile robots.

Smith et al. [6] present a method for automatically generating optimal robot paths satisfying high-level mission specifications. The motion of the robot in the environment is modelled as a weighted transition system. The mission is specified by an arbitrary linear temporal-logic (LTL) formula over propositions satisfied at the regions of a partitioned environment. The mission specification contains an optimizing proposition, which must be repeatedly satisfied.

Montemerlo et al. [7] present the architecture of Junior, a robotic vehicle capable of navigating urban environments autonomously. In doing so, the vehicle is able to select its own routes, perceive and interact with other traffic, and execute various urban driving skills including lane changes, U-turns, parking, and merging into moving traffic. The vehicle successfully finished and won second place in the DARPA Urban Challenge.

There is also lot of research devoted to path selection and trajectory optimization approaches. Adouane et al. [8] show a method based on elliptic trajectories to perform robot navigation, and Willms and Yang [9] present a robot path planning approach based on a dynamic propagation system.

Other approaches try to obtain optimal trajectories for manipulators: Constantinescu [10] presents a method to obtain smooth and time-optimal paths under some constraints; Gasparetto and Zanotto [11] present an approach to obtain trajectory plans based on an objective function which takes into account the integral of the squared jerk along the trajectories; Zanotto et al. [12] propose a methodology to compare trajectory planning algorithms for robotic manipulators, considering both the execution time and the squared jerk along the trajectory.

There are many other path planners found in the literature: Dolgov and Thrun [13] present a path planning algorithm that generates smooth paths for an autonomous vehicle settled on an unknown environment; Chang and Yamamoto [14] present a hybrid path planning based on Potential Field method and Voronoi Diagram approach whose aim is to concurrently navigate and perform map-building in a mobile robot.

Although lots of planning algorithms have been proposed over these years, a measurement of their performance only recently gained significant attention by the research community. Some examples of the efforts made to build common test environments are the collection of benchmark problems of the Algorithm and Application Group at Texas A&M University (https://pwww2.cse.tamu.edu/dsmft/benchmarks/) and the MOVIE project (http://www.cs.uu.nl/centers/give/movie/index.php), whose outcome is a database that contains many different test scenes that can be used to benchmark different motion planning techniques.

Calisi and Nardi [15] concentrate on pure-motion tasks: tasks that require moving the robot from one configuration to another, either as an independent subtask of a more complex plan or as a goal itself. After characterizing the goals and the tasks, they describe the commonly used problem decomposition and different kinds of modelling that can be used, from accurate metric maps to minimalistic representations. The contribution of their paper is an evaluation framework that they adopt in a set of experiments showing how the performance of the motion system can be affected by the use of different kinds of environment representations. In the same line, Calisi et al. [16] present a framework of benchmarks (MoVeME) (http://www.dis.uniroma1.it/calisi/index.php?page=moveme) for evaluating and comparing motion algorithms for autonomous mobile robots and vehicles. Karaman and Frazzoli [17] present an attempt to compare path planning algorithms from the quality point of view.

Many recent research papers address the problem of motion planning for self-driving vehicles, a context in which navigation evaluation methodologies become important. Werling et al. [18] deal with the trajectory generation problem faced by an autonomous vehicle in moving traffic. They propose a semireactive planning strategy that realizes all required long-term maneuver tasks (lane-changing, merging, distance-keeping, velocity-keeping, precise stopping, etc.) while providing short-term collision avoidance. Rastelli et al. [19] present a smooth trajectory generator computationally efficient and easily implementable, which considers Bezier curves and circumference parametric equations for a real vehicle. Moreover, this new trajectory generator reduces the control actions, generated with to a fuzzy controller.

## 3. Proposed Approach

The approach we present in this paper is mainly based on statistical measures for the trajectories performed. In this way, we first decide which set of features could be useful to measure the quality of a trajectory; then, we automatically select the most significant features among them; afterwards, we collect the selected features for a set of different trajectories, and finally we use these feature values to compare, from a statistical point of view, which of the used navigation techniques show a greater quality in the trajectories made.

The new approach works as follows.

(1) Decide the feature set which could help in the statistical measure of the trajectory quality.

Depending on the environment, goal, sensors provided by the robot, and the amount of possible dynamic obstacles expected, it is not supposed to be a unique set of features to be used. Nevertheless, the proposed approach could be used almost in any situation in which a navigation method has to be selected (semistructured environments, outdoor ones, UAV, submarine robots, etc.).

(2) Collect the values of these features for a set of trajectories.

It is noteworthy that the data can be obtained with the real robot in its appropriate environment or by a simulation software.

(3) Select the subset of features which maximizes the information.

This is the first thing to be done by a statistical analysis of the data. There are several ways to obtain this subset, but we have selected to use the Friedman test statistic [20], which is used as fitness value in an Estimation of Distribution Algorithm (EDA) [21] guided search. Friedman test has been found as a very sound method to compare the joint probability behaviour when different approaches are to be tested [22].

(4) Perform a statistical comparison using the selected features.

In this step, a statistical hypothesis test is carried out for each of the selected features to identify the statistically significant differences; obtained results are visualized graphically in the next step.

(5) Visualize the data.

We present a new approach to show the results of the tests and to decide, for each type of environment, which is the navigation technique that performs best.

Using this new approach an experiment has been performed in order to make a comparison between two global navigation algorithms. Next sections present the experimental design and the obtained results.

The self-driving vehicles framework is a context of interest for the proposed approach to be applied, due to its increasing interest during last years. Different kind of sensors, such as Kineck RGB-D [23], and different environments in which real-time trajectory calculation is needed have been presented in the literature [24, 25]. The approach introduced in this paper can be used as well in these scenarios; a specific feature selection is needed: the procedure to be used is the one presented in this paper.

## 4. Simulation Setup

We tested the proposed approach using two motion planners.

*(i) Fast Marching Square *(FM^2^). The Fast Marching Square (FM^2^) planner is a potential field based path planning algorithm whose main goal is to drive the robot to the goal in a smooth and safe way. The FM^2^ method is based on the way electromagnetic waves propagate in nature, because the potential and its associated vector field have good properties desired for the trajectory, such as smoothness and absence of local minima. The FM^2^ method makes use of the Fast Marching (FM) method, an efficient numerical algorithm to represent the front propagation proposed by Sethian [26]. It is a particular case of the Level Set methods [27], designed for problems in which the speed function never changes sign, so that the front is always moving forward or backward. The FM^2^ method is a two-step algorithm. In the first step, a wave is propagated using FM starting from the points representing the obstacles. The result of this step is a greyscale potential map where obstacles are black, and the farther a location is from an obstacle, the lighter it becomes. This map is known as a refraction index map because it can be seen as a varying refraction index environment where a new wave will draw a trajectory. In the second step, a new wave is emitted from the goal point over the refraction index map using FM. This way a unique field is obtained and its associated vector field is attracted to the goal point and repulsed from the obstacles. Finally, the shortest time trajectory is calculated in this potential surface by using the gradient method.

*(ii) WaveFront.* This planner shares some similarities with the FM^2^ method in the way the wave is propagated. Besides, it is offered as a planner driver within the Player/Stage software set. The WaveFront planner works as follows: it first creates a configuration space of grid cells from the map that is given, treating both occupied and unknown cells as occupied. Then, based on their distance to the nearest obstacle the planner assigns a cost to each of the free cells (the nearer the obstacle, the higher the cost). When the planner is given a new goal, it starts from the goal cell and assigns plan costs to the cells as it expands like a WaveFront. The plan cost in each cell is dependent on its distance from the goal, as well as the obstacle cost assigned in the previous step. Once the plan costs for all the cells have been evaluated, the robot can simply follow the gradient of each lowest adjacent cell all the way to the goal.

Usually, global planners should be combined with some obstacle avoidance strategy or local planner in order to cope with dynamic environments and reduce the need and hence the computational payload of replanning when changes in the environment make the original path unfeasible. Therefore, we used the stated global planners in combination with two local planners (also available within Player/Stage): the Vector Field Histogram Plus (VFH+) [28] and the Nearness Diagram (ND) [29]; the local planners were used in order to see how the local method affects the performance of the whole system.

Tests were made in simulation environments as the first step towards the application in the real world. Simulation tools allow analysis of the behavior of the methods with respect to a wide spectrum of situations and collect a great amount of data for statistical analysis. The stage simulator of the Player/Stage package was chosen for this experiment because it is widely used by the scientific community and is also included in some Linux distributions. Tests were made using two simulated robots, the popular Pioneer 2DX and our Segway RMP 200.

### 4.1. Environments Used

**Figure 1:**
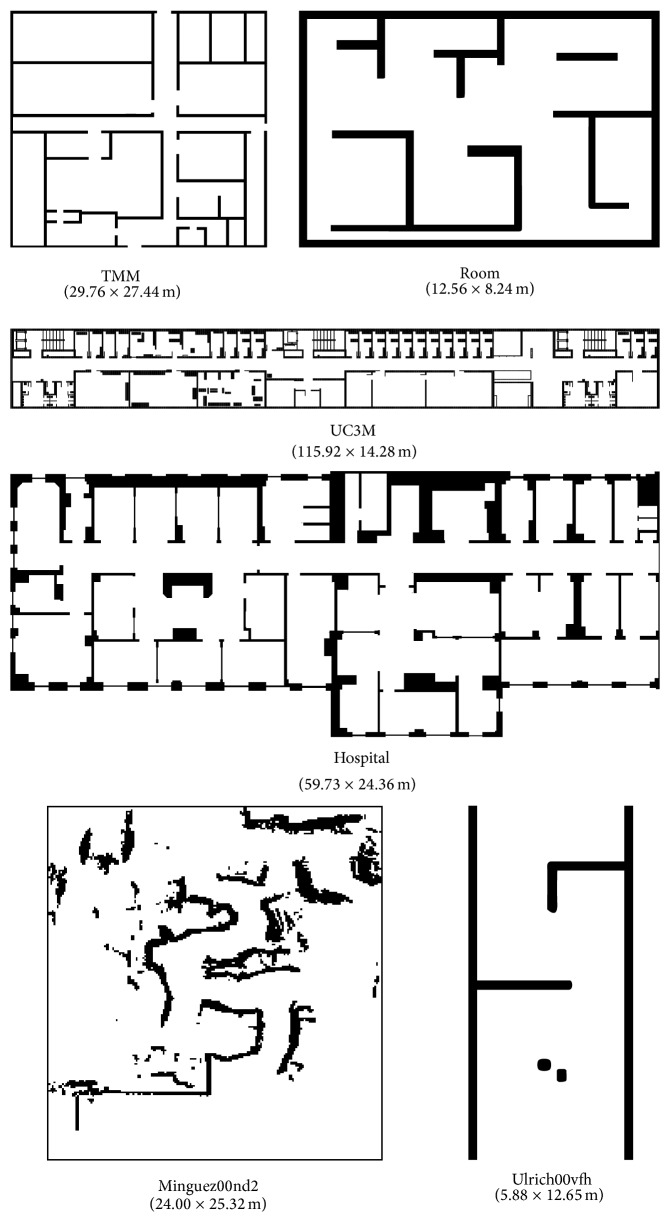
Used environments.

To test the proposed approach, six simulation environments were used with the Player/Stage software. Three of these environments were created by us (a synthetic test room, an office indoor environment of Carlos III University and Teknikers laboratory) and the other three were taken from the “Mobile robots and Vehicles Motion algorithms Evaluation” (MoVeME) benchmark database (hospital, minguez00nd2, and ulrich00vfh).  Figure 1 shows these environments, as well as the size of each of them. The idea behind the selected environments is to test the motion methods under typical and critical situations. Thus, room, uc3m, tmm, and hospital correspond to typical office-like indoor scenarios whereas minguez00nd2 and ulrich00vfh are critical situations examples given in past research papers [28, 29].

### 4.2. Trajectories

The data collection is made based on ten trajectories performed by the robots in each of the six environments using FM^2^ and WaveFront as global planners and in both of them the local navigation algorithms VFH+ and ND. In order to ensure the soundness of the experiments, the robot start position was fixed in each environment and 10 goal positions were randomly selected. For comparison purposes the same targets were commanded to both FM^2^ and WaveFront motion planners.

**Figure 2:**
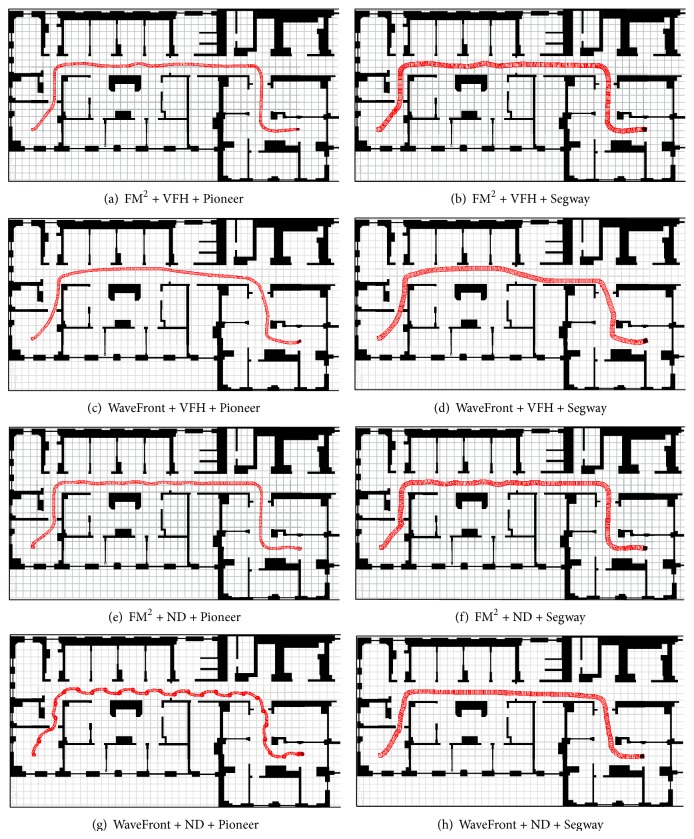
Hospital: examples of performed trajectories.

Figure 2 shows an example of the different trajectories performed in order to achieve a randomly obtained goal.

### 4.3. Data Collection

**Figure 3:**
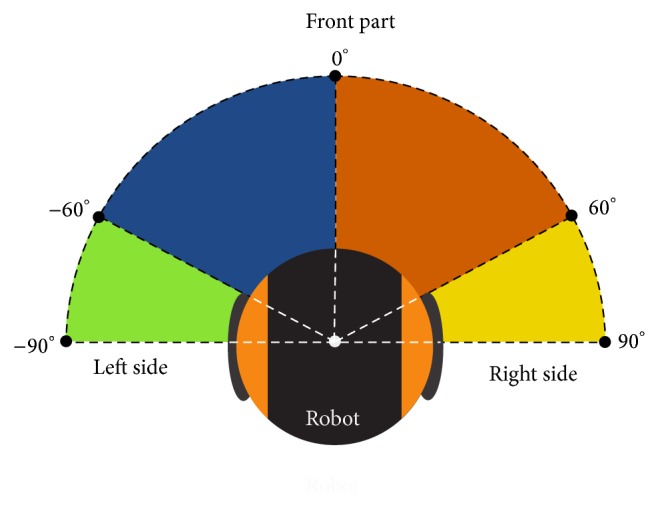
The angle areas used for data collection.

It seems logical to characterize the trajectories using the data provided by the sensors on the robot. In the experiment performed, we use a laser device as leading sensor for the navigation system, and hence the laser readings are used for comparison purposes. First of all, four different regions of the laser reading area were defined (see Figure 3):

- Left side angle 1, from −90 to −60 degrees.
- Left side angle 2, from −60 to 0 degrees.
- Right side angle 2, from 0 to 60 degrees.
- Right side angle 1, from 60 to 90 degrees.

Once these regions were defined, the data collection consisted of measuring, for each trajectory, the 11 features shown in Table 1.

As the proposal is to compare two global navigation algorithms, we performed 10 random trajectories in each of the 6 environments, using two robots and two local navigation algorithms, which sum up a total of 10 × 6 × 2 × 2 = 240 trajectories, and the values of these 11 variables were collected for all the trajectories performed.

It is worth mentioning that, in the experiments carried out, some of the trajectories (48, all of them with the WaveFront planner) did not arrive to the commanded final location. Although this fact could give some information for the comparison, we decided to remove the data of the failed trajectories. The reason to do so is that it could be considered as a specific problem of the used environment/goals, and we aim to present a general comparison approach in any situation. Nevertheless, in any comparison to be done, the amount of nonending paths for each of the compared approaches is a matter to be taken into account, as it could indicate that one of the used methods outperforms the other in the amount of completed trajectories, even though in completed ones the performance of the worst approach seems to be better.

### 4.4. Feature Subset Selection

Not all the initially selected features are going to be used for the performance comparison activity; a feature selection is made previously, in order to obtain the feature subset which gives more information about the differences between the used global navigation algorithms. The selection is based on the Friedman test value (see the Appendix), while the search is made using and evolutionary algorithm called Estimation of Distributions Algorithm (EDA), as explained below.

#### 4.4.1. Searching Method

Feature Subset Selection (FSS) can be viewed as a search problem [30], with each state in the search space specifying a subset of the possible features of the task. Exhaustive evaluation of possible feature subsets is usually infeasible in practice due to the large amount of computational effort required.

Estimation of Distribution Algorithms (EDAs) have successfully been developed for combinatorial optimization [31, 32]. They combine statistical learning with population-based search in order to automatically identify and exploit certain structural properties of optimization problems. EDAs typically work with a population of candidate solutions to the problem, starting with the population generated according to the uniform distribution over all admissible solutions. The population is then scored using a fitness function. This fitness function gives a numerical ranking for each string, so that the higher the number the better the string. From this ranked population, a subset of the most promising solutions are selected by the selection operator. An example selection operator is truncation selection with threshold *τ* = 50%, which selects the 50% best solutions. The algorithm then constructs a probabilistic model which attempts to estimate the probability distribution of the selected solutions. Once the model is constructed, new solutions are generated by sampling the distribution encoded by this model. The new solutions are then incorporated back into the old population, possibly replacing it entirely. The process is repeated until termination criteria are met (usually when a solution of sufficient quality is reached or when the number of iterations reaches some threshold), with each iteration of this procedure usually referred to as one generation of the EDA.

### 4.5. Selected Attributes

After the search process, we obtain as the most valuable feature subset that composed by the variables {*X*_3_, *X*_4_, *X*_7_, *X*_8_, *X*_11_} (see Table 2), that is, the length of the trajectories, the mean of the five minimum values (right and left), and the mean of the front laser readings (right, left). These are the variables to be used in the experimental phase below, to establish a comparison between different global navigation algorithms, and to be able to select the most suitable navigation method for each environment. As a result, the remaining variables are discarded by the Friedman test guided EDA selection approach.

### 4.6. Feature Based Comparison: One by One

First of all, we compare the selected attributes one by one, in order to determine whether there are any differences between the feature values obtained by each of the compared methods. A nonparametric statistical test called Wilcoxon Rank test [33] is used to this end; the Wilcoxon test was developed to analyze data from studies with repeated-measures and matched-subjects designs. Obtained variable values are used in the comparison.

#### 4.6.1. Wilcoxon Signed Rank Test

Suppose we collect 2*n* observations, two observations of each of the *n* subjects. Let *i* denote the particular subject that is being referred to and let the first observation measured on subject *i* be denoted by *x*_*i*_ and the second observation be denoted by *y*_*i*_. For each *i* in the observations, *x*_*i*_ and *y*_*i*_ should be paired together.

Let *D*_*i*_ = *x*_*i*_ − *y*_*i*_, ∀ *i* = 1,…, *n*. 

*Main Idea.* If neither condition has an effect, then not only should the differences be equally distributed on either side of 0, but also the distance from 0 should be the same on either side. Consider the following:

1. let *R*_*i*_ be the rank of |*D*_*i*_| (absolute value of difference);
2. restore signs of *D*_*i*_ to the ranks → signed ranks;
3. calculate either
4. *W*_+_ = sum of ranks with positive signs,
5. *W*_−_ = sum of ranks with negative signs;
6. *H*_0_: distribution of *D*_*i*_ is symmetric about 0;
7. *H*_1_: we can not say that distribution of *D*_*i*_ is symmetric about 0.

It can show (if no ties)

(1) $$\begin{array}{c} E_{H_{0}}\left(W_{+}\right)=\frac{n\left(n+1\right)}{4},\\ \text{Va}{\text{r}}_{H_{0}}\left(W_{+}\right)=\frac{n\left(n+1\right)\left(2n+1\right)}{24},\\ Z=\frac{W_{+}-\left(n\left(n+1\right)\right)/4}{\sqrt{n\left(n+1\right)\left(2n+1\right)/24}}\sim^{\text{approx}}N\left(0,1\right). \end{array}$$

Using this nonparametric test we can compare couples of values obtained for the values of some variable (feature) by two different methods.

Table 3 shows the obtained *P* value results of the Wilcoxon test using all the data collected in the performed trajectories. The first row presents the results obtained using all the collected data (six environments, two robots, two navigation algorithms for each of the compared methods). The rest of the rows show the results obtained for each of the environments. As it can be seen, differences appear in some of the selected variables, but it is still needed to visualize which of the methods obtains better result (i.e., which method is the difference for). With this goal in mind, a graphical data visualization has been depicted in order to obtain a more clear view of the obtained results.

### 4.7. Data Visualization: All the Selected Variables in One Single Picture

The proposed method to visualize the obtained results after applying the Wilcoxon test is a graphical one. More precisely, it is a polygon in which the obtained results for each individual test are shown. As five are the selected variables for the proposed comparison, pentagons are used for this case, but the number of sides could vary, as it depends on the proposed experiment, resulting in general in a polygon of *n* sides, where *n* is the number of selected variables.

The selected value to be shown graphically is the 1 − *P* significance of the Wilcoxon test. The reason is that the aim of the comparison is to stress the differences—if any—between the compared two approaches. If we directly plot *P* values, the appearance of differences results in values placed in the centre of the pentagon, making visualization more difficult and thus the finding of clear conclusions. By contrast using 1 − *P*, if there exist differences they are placed close to the perimeter and the figure results more visual.

In this way, and aiming at highlighting the significance of the test made over each edge, we inscribe an internal pentagon which has a 0.8 length in each edge—that is, a *P* value of 0.2. The idea is that the values over that inscribed pentagon are those to be taken into consideration for the comparison. It could be seen as a “trajectory” of the values over 0.8, which show the differences.

**Figure 4:**
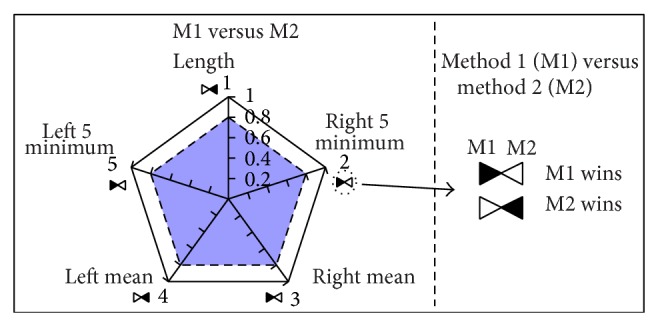
Developed visualization method.

Figure 4 shows the defined visualization approach named as* polygraph*; the pentagon is shown in the left side. The order of the five edges has been set for clarity, and therefore the right edges correspond to the 5 minimum values mean (up) and −60 to 0 values mean (bottom) while the left edges refer to the 0 to 60 values mean (bottom) and 5 minimum values mean (up); the central edge depicts the differences between the length of the performed paths. In addition, differences could favor the first or the second method. For instance, if our purpose is to reach the goals as soon as possible, shortest trajectories are desired. To clarify that, as shown in the right side of Figure 4, we draw a shape composed by two triangles, in which the filled one indicates the winner method.

**Figure 5:**
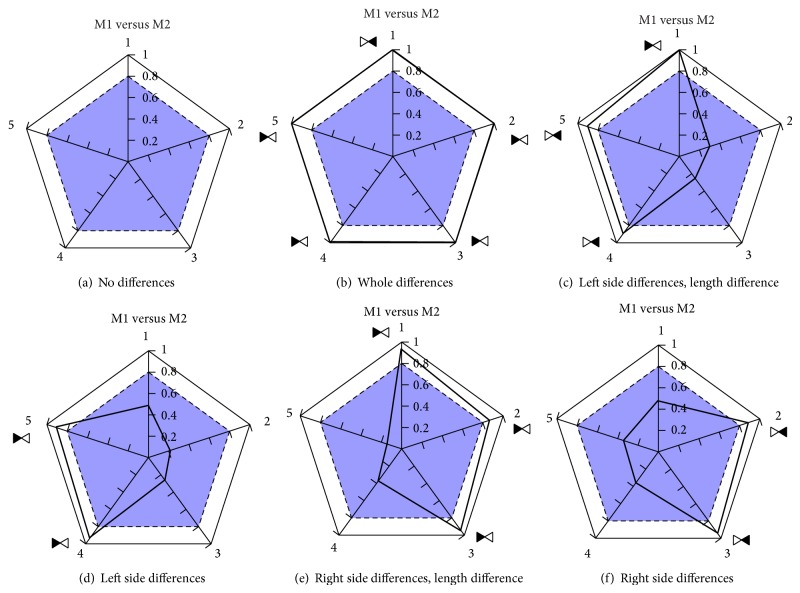
Pentagon like comparisons.

Therefore, what is important to take into account is the surface obtained (both in area and in shape) after the five edge values are given.  Figure 5 shows several pentagon shapes that can be obtained depending on the results of the comparison. Hence, the pentagon should appear clean if no differences are identified (Figure 5(a)), full signigicant differences appear in all the variables (Figure 5(b)), and the different shapes it can take depending on the source of the differences, as shown in Figures 5(c), 5(d), 5(e), and 5(f).

Regarding the differences which appear as it has been stated above, it has to be shown which of the compared methods obtains better results. For that purpose a symbol has been defined as shown in Figure 5. The defined symbol is only used when differences are present, so it is only drawn in those 1 − *P* values over 0.8. For instance, Figure 5(b) is an example in which one of the compared methods (the first one, M1) outperforms on all aspects of laser readings, while it obtains worse result for the length of the performed trajectories. In this case, the selected approach would be probably M1; the same happens with Figure 5(e), where only right side differences appear in favor of M1, but M2 performs better with respect to the length of the trajectories (in some few cases, the user could select M2, mainly if all the trajectories made with it reached the goal).

More clear differences are shown in Figure 5(d), in which M1 would be selected as the best method, whereas the results obtained in Figure 5(f) indicate that M2 is the approach to be used. Finally, the decision to be made under Figures 5(a) and 5(c) is not clear at all: Figure 5(a) results indicate the same behaviour of the compared methods, so other parameters are to be taken into consideration (price, computational load,…). On the other hand, the decision in the case of Figure 5(c) will completely depend on the specific task to be done (M1 behaves more secure; M2 optimizes the trajectories).

## 5. Experimental Results

Using these* polygraphs* (pentagons in the experiments performed, which can be used in a wide range of environments) we have carried out a comparison between the two global navigation algorithms: FM^2^ and WaveFront. The idea is to select the best algorithm in general, or the more appropriate one for a given environment.

### 5.1. All Collected Data

**Figure 6:**
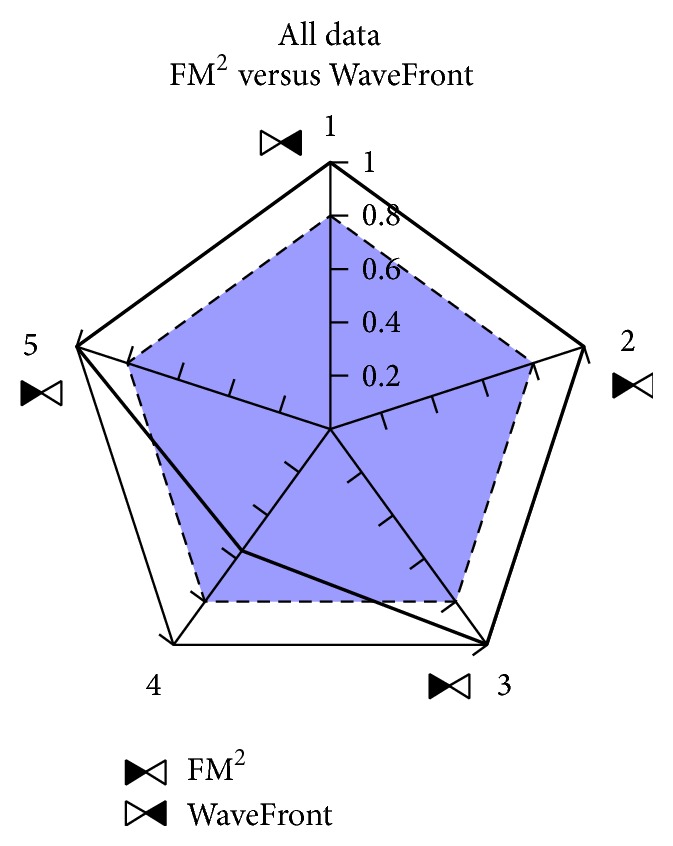
All data results.

In order to show the obtained results, we present in Figure 6 the pentagon which contains the overall Wilcoxon related results when all the environments, robots, and local navigation systems are considered. As it can be seen, differences appear over the threshold established in four of the five edges; three of the edges indicate a better performance of FM^2^ against the only one that favors WaveFront (shorter lengths are obtained), and hence FM^2^ would be the selected one. At this point, if the user is not sure about the selection to be made, it would be necessary to look at the failed trajectories—if any—which in our case strengthens the FM^2^ quality.

It is interesting to point out that the selection has been made based on the total number of edges that indicate a better performance, giving the same weight to all of them. If the task for which the method is going to be used imposes some requirements, one could consider to give more weight to the features that better represent those requirements. For example, if our main goal is to reach a destination as soon as possible, we would choose WaveFront instead of FM^2^, because it generates shorter trajectories (see edge 1 of the pentagon), at the expense of losing safety (see edges 2, 3, and 5 of the pentagon).

### 5.2. Environments One by One

**Figure 7:**
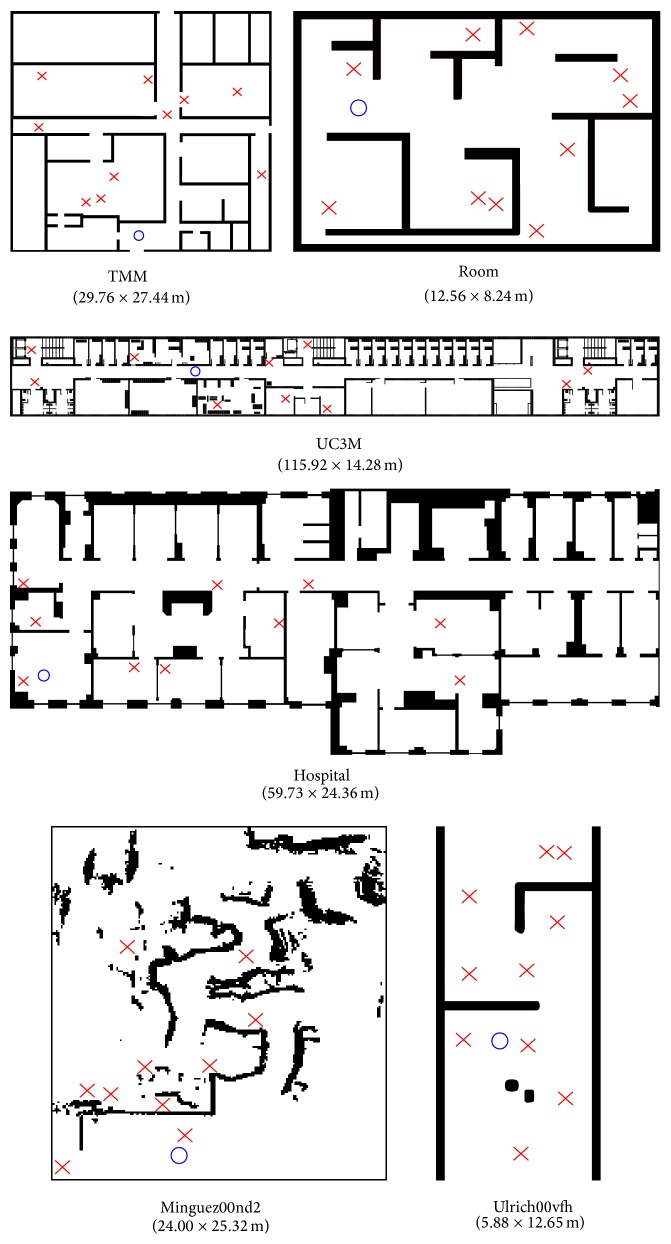
Trajectories starting and end positions.

**Figure 8:**
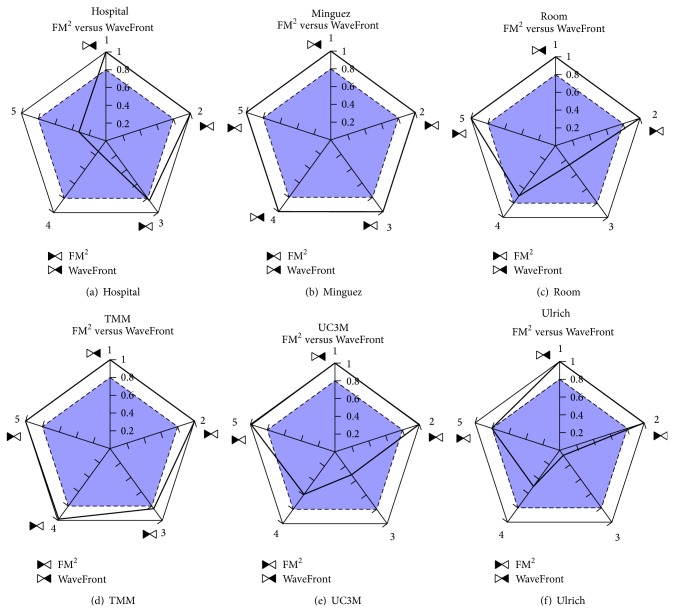
Results for each environment.

As stated before, for each environment an initial position has been fixed, and 10 goal positions have been randomly selected.  Figure 7 shows the starting points (with a blue circle) and the goal points (with a red “X”) used in each environment. The experimental results obtained with the collected data in each environment are shown in Figure 8. A thorough look at the obtained pentagons shows that length differences appear in all the environments (WaveForm is better from that point of view), but the other differences found are better for the FM^2^ approach. In this way, right side differences appear in the Hospital environment (Figure 8(a)), which probably are due to the random trajectories selected (see Figure 2); in Room, TMM, UC3M, and Ulrich environments differences in left and right sides appear (except in the right side 5 minimum mean) which indicates that the distance to obstacles from both sides is larger when using the FM^2^ algorithm; in the same way, with respect to Minguez environment, differences appear in the left side readings mean and in the right 5 minimum mean in favor of the FM^2^ algorithm.

## 6. Conclusions and Future Work

A new approach to perform robot trajectory comparison is presented in this paper; the method could be applied to any kind of trajectory comparisons, both in indoor and outdoor environments and also in simulated and real situations. As an example, the approach is applied to analyse trajectories made by two navigation algorithms (FM^2^ and Waveform) in six different simulation environments, with two different robots and using two different local planners as well.

The trajectory comparison method is based on well known statistical tests and thus provides a sound mathematical foundation, and it can help to decide the navigation algorithm to use, the most suitable robot to a particular environment, or to select a combination of methods (for instance, local navigation and global navigation) that are preferred for the goal the robot is programmed for.

It is also important to note that the method offers a visual way to compare different aspects related to the trajectories performed. The polygonal structures obtained give us an image of the relationships of the edges among the selected features. This fact could be used by users to understand better the meaning of the obtained polygons (pentagons in the experiments performed).

In the near future a set of experiments are planned with real robots in real outdoors environments; to deal with the sensors data, a DGPS system with a 2 cm localization error is to be used, combined with a laser sensor and a camera.

As future work, this statistical comparison has to be extended to other environments in which the application is not direct (or, to be more precise, it can be applied but maybe not so well visualized). A set of experiments has to be done with 3D environments (UAV, submarines) in order to improve the way to visualize the 3D comparisons.

## Figures and Tables

**Table 1:** Collected data.

Feature	Data
*X*_1_	Right angle region 1
*X*_2_	Left angle region 1
*X*_3_	Right angle region 2
*X*_4_	Left angle region 2
*X*_5_	Right side minimum laser range value
*X*_6_	Left side minimum laser range value
*X*_7_	Right side minimum five laser range values mean
*X*_8_	Left side minimum five laser range values mean
*X*_9_	All reading mean
*X*_10_	All reading minimum five laser range values mean
*X*_11_	Length of the performed path

**Table 2:** Selected features.

Feature	Data
*X*_3_	Right angle region 2 (0 to 60 degrees)
*X*_4_	Left angle region 2 (−60 to 0 degrees)
*X*_7_	Right side minimum 5 values mean
*X*_8_	Left side minimum 5 values mean
*X*_11_	Length of the performed path

**Table 3:** Wilcoxon test results.

	*X*_3_	*X*_4_	*X*_7_	*X*_8_	*X*_11_
All	1	8.2*E* − 19	0.9977	0.4363	0.9999

Hospital	0.9999	4.2*E* − 05	0.8318	0.1469	0.3192
Minguez	0.9999	0.0013	0.9999	0.9982	0.9998
Room	0.9999	0.9999	0.2658	0.7	0.9999
TMM	0.9999	0.9999	0.8364	0.9819	0.9999
UC3M	0.9999	0.9941	0.3182	0.5916	0.9895
Ulrich	3.1*E* − 07	0.9966	0.0696	0.5015	0.8032

## Appendix

### Friedman Test

The Friedman test [20] is used to guide the selection or, to be more precise, the obtained Friedman test value for a set of variables; Friedman test is a nonparametric randomized block analysis of variance. That is to say, it is a nonparametric version of a one-way ANOVA with repeated measures. This means that while a simple ANOVA test requires the assumptions of a normal distribution and equal variances (of the residuals), the Friedman test is free from those restrictions. This test has been used in the literature when different collections of multiple variable data are to be compared [22] and hence it has been found appropriate for the purpose of this research.

The hypotheses for the comparison across repeated measures are

(i) H0: the distributions (whatever they are) are the same across repeated measures;
(ii) H1: the distributions across repeated measures are different.

The Friedman statistic (M) is calculated by using

(A.1)M=12nkk+1∑‍Rj2-3nk+1,

where (i) k is number of columns (features in our case), (ii) n is number of rows (trajectories), and (iii) $R_{j}$ is sum of the ranks in column j .

The test statistic for the Friedman test is a Chi-square with [(number of repeated measures) − 1] degrees of freedom.

This statistic is used to select among initial set of features those which result in a bigger difference between the methods to be compared. Hence, a bigger value of the Friedman Test statistic is preferred.
